# Strategies for Success: Assessing Student Perspectives on the Impact of Step 1 Pass/Fail

**DOI:** 10.1007/s40670-025-02434-4

**Published:** 2025-06-18

**Authors:** Michaela J. Derby, Callie Olson, Pasquale Manzerra, Lori A. Hansen

**Affiliations:** USD Sanford School of Medicine, Vermillion, SD USA

**Keywords:** Step exam, Pass/Fail, Curriculum, Student, Academic advising, Mental health

## Abstract

The United States Medical Licensing Examination Step 1 Exam became pass/fail in January 2022 with an objective of reducing the test-taking pressure for medical students. The impact of Step 1 pass/fail on student experiences and outcomes is unknown. We responded by surveying the classes of 2024 and 2025 with five Likert-scale questions to assess the impact of curriculum, mental health, and academic advising/preparation on the Step 1 pass/fail experience. We surveyed students’ experiences with counseling services, advising about mental health, weekly quizzes, completing pre-clerkship curriculum in 1.5 years, the order in which our pre-clerkship courses were completed, and time dedicated to study for Step 1. Students were surveyed on their experience with peer guidance/advice, student panels, and advising for exam readiness. Open-response questions assessed individualized preparation. Results demonstrate that students are facing adversity in mental health that may be mitigated with frequent academic advisor meetings, increased access to mental health counselors, and reduced isolation. Students desire more National Board of Medical Examiners test preparation. Results also indicate that the pre-clerkship curriculum may benefit from supplemental student-led Step 1 preparation and structured peer mentoring for Step 1. Our results may benefit other schools looking to implement change and improve their medical education with emphasis on those anticipating moving to a 1.5-year curriculum. If needed, medical schools may consider soliciting a similar survey to assess their own medical education’s impact on students with Step 1 now being pass/fail.

**Supplementary Information:**

The online version contains supplementary material available at 10.1007/s40670-025-02434-4.

## Background

The United States Medical Licensing Examination (USMLE) Step 1 Exam began reporting in pass/fail starting in January 2022 [1, 2]. Before the reporting change, students faced immense pressure to receive a numerically high Step 1 score to enter competitive specialties [3, 4]. A numerical Step 1 score negatively impacted student mental health and deemphasized clinical education that is not directly tested on the Step 1 exam [3, 4]. Overall, anxiety is similar between cohorts that took Step 1 pass/fail compared with a numerical score [5]. However, it has been reported that there is a difference in anxiety at a specific timepoint between the groups within the pre-clerkship curriculum. When medical students took Step 1 pass/fail, they experienced less stress in the months leading up to their dedicated study period (dedicated period is typically 4–6 weeks to focus solely on studying for the Step 1 exam [6]) than those who took the exam for a numerical score. As students entered the dedicated study period, whether they were taking the exam for a numerical score or pass/fail, the stress normalized with intense studying leading to isolation, stress, exhaustion, and burnout [7–9]. With Step 1 now pass/fail, student anxiety and pressure to score highly on Step 2 have increased to match into competitive specialties [10].

Although mental health for students taking Step 1 pass/fail is improved before entering the dedicated study period, there remains heightened stress, specifically in the month leading up to the Step 1 exam date and a concerning decline in pass rates for all first-time test takers [6, 7]. Between the years of 2021 and 2022, the US/Canadian Medical Doctor (MD) pass rates decreased by 3%, Doctor of Osteopathic Medicine (DO) by 5%, and non-United States (US)/Canadian schools by 8% [11]. Accompanying Step 1 moving to pass/fail, the National Board of Medical Examiners (NBME) increased the passing score of Step 1 from 194 to 196 in 2022 [12]. The good intentions of NBME making Step 1 pass/fail bring forth uncertainties like declining pass rates and continued stress, burnout, and anxiety in medical education that must be assessed to help better meet the mental health needs of students in the new pass/fail era [6].

Studying for in-house exams is a concurrent process for many medical students while also balancing the demands of preparing for Step 1 with outside resources. Students at Brown University are not only using lectures to study, but also relying heavily on outsourced resources. At Brown University, 84% of students report using institutional medical school lectures, 84% use Boards and Beyond lectures, 65% use Anki flashcards, 55% use Sketchy videos, 36% use Pathoma lectures, 74% use First Aid, and 72% use the USMLE Rx Question Bank [13]. The usage of outside resources alongside traditional lecture style, known as a “parallel curriculum”, is an onerous yet critical part of education for the modern medical student. Studies indicate that 70% of students experienced anxiety and stress due to the misalignment between the medical school curriculum and USMLE Step 1 content [13]. One expert viewpoint on medical student curriculum states that if the goal is for medical students to learn curricula efficiently, insights as to how medical students apportion their study time should inform curricula decisions [14], e.g., recommending flashcards that correspond to lectures, designing more curricula content in the form of flashcards as opposed to slides and handouts, and even decreasing the number of faculty-delivered lectures. Only 48% of medical schools integrate external resources into the pre-clinical curriculum [14]. Formal incorporation of these resources for the independent learner into traditional teaching methods to eliminate redundancies is an expert suggested path forward that has already proven to be effective in Step 1 success and may lend to further consideration with the transition to Step 1 pass/fail [6, 14].

The implications of the Step 1 pass/fail reporting format on medical student experience and outcomes are not well understood. Medical schools, particularly the many schools anticipating moving from a 2-year pre-clerkship curriculum to a 1.5-year pre-clerkship curriculum due to Step 1 moving pass/fail, may benefit from understanding how a 1.5-year pre-clerkship medical education program is perceived by medical students with this drastic change to Step 1 [14]. As schools navigate this change in their medical education, it will be important to consider student needs prior to making big changes to pre-clerkship timelines to improve outcomes for their medical students. The purpose of our study was to understand student perspectives on the impact of Step 1 pass/fail on mental health, curriculum, and academic advising at our medical school. Our findings may either be directly implemented or used as a guide for survey creation with some emphasis on medical schools considering evolving to a 1.5-year pre-clerkship curriculum, like our own, to strengthen and adapt their medical education to the modern self-directed student learner.

## Materials and Methods

A twenty-three question survey was created to assess the University of South Dakota Sanford School of Medicine medical student perspective on preparation in mental health, curriculum design and content, and academic advising with Step 1 moving to pass/fail. The survey was accessed through an embedded hyperlink in an email sent to students explaining the purpose, time to complete, and anonymity. The survey was initially sent September 11, 2023, and we accepted responses until September 29, 2023. Emails containing the survey hyperlink were sent a total of four times. The email containing the survey hyperlink explained that informed consent was implied by choosing to complete the survey. Students in the class of 2024 and 2025 completed the survey over 1.5 years and 8 months after taking Step 1, respectively. We surveyed students who completed Step 1 primarily in January 2022 and 2023 after completing Pillar 1, defined as the first 1.5 years of pre-clerkship curriculum at our school. During Pillar 1, students complete pre-clerkship courses including those covering the basic sciences and foundations for clinical medicine. Pre-clerkship courses were completed in the following order: Medical Foundations 1, Medical Foundations 2, Skin-Musculoskeletal, Neuro, GI-Hepatobiliary, Blood/Hemo/Lympho, Cardiovascular, Renal-Urinary, Respiratory, and Endocrine-Reproduction. After passing all courses in the first 18 months of medical school, students are eligible to take the Step 1 exam before entering Pillar 2, the 12-month clinical clerkship courses. Lastly, students enter Pillar 3 for electives and required rotations including research, away rotations, sub-internships, and more school-required and specialty-specific courses. This Pillar lasts 16 months. Medical students have access to mental health resources at our school during all Pillars. Mental health resources at our school consist of meetings with a Chief Wellbeing Officer, free, unlimited access to a counselor dedicated to medical students, and wellness committee events. The school Chief Wellbeing Officer advises about wellness and meets with students in both group and one-on-one settings throughout Pillar 1. The survey was sent to every medical student in the class of 2024 (*n* = 62) and 2025 (*n* = 63), and all were eligible to participate.

The survey questions included three demographic questions (gender, year graduated, and when the student took the USMLE Step 1 Examination), five matrix-style questions with Likert-scale responses about their experience with Step 1 pass/fail (overall preparation, mental health, curriculum, academic advising, and negative impacts on studying for Step 1 the semester prior to their exam date), and seven questions regarding preparation (time spent studying in Pillar 1, ideal time studying in Pillar 1, time per week in the summer, ideal time studying in the summer, study tactics, when it was hard to study, time spent per week in blocks, factors impacting studying). The mental health question asked about counseling services, wellness advising, and wellness committee events. The curriculum question asked about weekly quizzes, completing pre-clerkship in 1.5 years, test question similarity to the National Board of Medical Examiners (NBME) style questions, completing comprehensive basic science subject exams (CBSE) and NBMEs in Pillar 1, integrating clinical skills into pre-clerkship curriculum, content taught versus content tested, lecture style, supplemental instruction review sessions, the order in which our pre-clerkship courses were completed, and sufficient dedicated and Pillar 1 time to study for Step 1. The academic advising section asked about peer guidance and advice, student panels, advising for exam readiness, 1:1 meetings with the Medical Education Learning Specialist, tools to determine specialty fit, school-offered career advising, and promoting resume competitiveness. The question about negative impacts on studying for Step 1 asked about Step 1 being pass/fail rather than scored, class rank, achieving good grades, understanding course content, receiving a passing grade, extracurriculars, time with family, and mental health. The full survey and entirety of subcategory questions are included in supplementary information 1.

Additionally, there were three open-response questions that asked for general comments on curriculum, mental health, and academic advising. We summarized and quantified open responses that were shared by multiple students with similar perceptions. This study was exempted from the University of South Dakota Institutional Review Board (IRB-24–61).

## Results

We had 67 responses out of 125 total students that were sent the survey with an overall response rate of 53.6%. Because responses to all questions were not required for completion of the survey, the total number of responses varied for each question. The class of 2024 accounted for 50.75% (*N* = 34/67) of the total responses, and the class of 2025 accounted for 49.25% (*N* = 33/67). Males comprised 47.76% (*N* = 32/67) of responses, and females were 52.24% (*N* = 35/67). Students felt they had underprepared the summer preceding their Step 1 examination. The mean time (± SE) studied in the summer was 5.4 (± 0.9) hours weekly. Students on average would have liked to have studied 9.3 (± 1.2) hours weekly. Study patterns varied among students surveyed in the 6 months leading up to their Step 1 examination date. Twenty-five percent (*N* = 14/57) studied a consistent number of hours each week, 35% (*N* = 20/57) ramped up hours or questions each week, 33% (*N* = 19/57) inconsistently studied, and 7% (*N* = 4/57) did not study. A substantial majority of students (82%; *N* = 47/57) reported that understanding content taught in the pre-clerkship curriculum either moderately, very much, or extremely negatively impacted their motivation to study for Step 1. A substantial majority of students (82%; *N* = 47/57) reported that achieving good grades either moderately, very much, or extremely negatively impacted students’ motivation to study for Step 1. Only 14% (*N* = 8/57) of students responded that Step 1 moving to pass/fail had a very much or extreme negative impact on their motivation to study for Step 1.

### Mental Health

A substantial majority of students (80%; *N* = 53/66) were neutral or agreed that the mental health services at our school helped them prepare for Step 1 pass/fail. A majority of students agree or strongly agree that school-offered counseling services (63%; *N* = 41/65) and school advising about wellness (57%; *N* = 37/65) supported their mental health with Step 1 pass/fail (Table 1). A total of twenty-eight open responses were collected from the mental health section. Student open responses supported the survey data, as two students commented on how the school-offered counseling services and six commented on how one-on-one meetings with faculty were among the best mental health support they received. One response noted that the counselor was very helpful, but hard to schedule an appointment with due to being overbooked. A substantial majority of students (77%; *N* = 50/65) were neutral or agreed that the wellness committee events were helpful (Table 1).

**Table 1:** Percentage of respondents who felt the school adequately supported their mental health in the following categories with Step 1 being pass/fail survey out of sixty-five responses

Mental health categories	Strongly disagree	Disagree	Neutral	Agree	Strongly agree
Counseling services	0	9.2	27.7	36.9	26.2
Advising about wellness	3.0	12.3	27.7	43.1	13.9
Wellness committee events	4.6	18.5	35.4	29.2	12.3

Thirteen of the student open responses indicated that they struggled with mental health. Three students described it as either the worst mental health they have experienced or the worst mental health experienced in medical school. Two students found it isolating, six found it to be a more challenging mental health effort than expected, and two wish they would have started studying earlier. Six students commented that having faculty check in on progress and concerns was supportive of mental health. One student commented that the day after the exam was their lowest point with mental health.

### Curriculum

A majority of students (61%; *N* = 40/66) agreed or strongly agreed that the curriculum prepared them well overall for Step 1 being pass/fail. A substantial majority of students agreed or strongly agreed that they were well prepared for Step 1 pass/fail due to the use of weekly quizzes (71%; *N* = 42/59) and completing Pillar 1 in 1.5 years (83%; *N* = 49/59) (Table 2). A total of thirty-seven open responses were collected from the curriculum section. Students reinforced these findings in the open-response section, with five students commenting that the curriculum was comprehensive and that weekly quizzes kept students accountable to studying.

**Table 2:** Percentage of respondents who felt the following aspects of the school’s curriculum adequately prepared them for Step 1 being pass/fail out of fifty-nine responses

Curriculum categories	Strongly disagree	Disagree	Neutral	Agree	Strongly agree
Weekly quizzes	6.8	11.9	10.2	49.2	22.0
Completing pillar 1 in 1.5 years	0.0	8.5	8.5	54.2	28.8
Test question similarity to NBME-style questions	10.2	28.8	17.0	25.4	18.6
Completing CBSEs after Medical Foundations 2, Blood block, and Endocrine block	1.7	8.5	18.6	44.1	27.1
Completing a NBME after each block	1.7	3.4	20.3	39.0	35.6
Integrated clinical skills into pre-clerkship curriculum	5.1	13.6	28.8	35.6	17.0
Order in which the blocks were completed (i.e., MF1 > MF2 > Skin/Msk > neuro)	0.0	3.4	15.3	52.5	28.8
Content taught in class versus content tested on Step 1	11.9	23.7	25.4	30.5	8.5
Delivery of Basic Science content in class (i.e., lecturing styles)	3.4	13.6	23.7	44.1	15.3
Sufficient time during Pillar 1 to study for Step 1	6.8	35.6	8.5	35.6	13.6
Sufficient dedicated time to study for Step 1	3.4	6.8	5.1	47.5	37.3
Supplemental Instruction Review Sessions	5.1	17.0	32.2	33.9	11.9

A substantial majority of students agreed or strongly agreed that they were prepared for Step 1 being pass/fail due to completing three CBSE assessments throughout pre-clerkship (71%; *N* = 42/59) years and completing a customized NBME assessment after each block (75%; *N* = 44/59) (Table 2). In open responses, eight students desired more NBME practice questions, and eleven wanted the in-house weekly quizzes to follow NBME-style questions and content more closely.

A substantial majority of students agreed or strongly agreed that they were prepared for Step 1 being pass/fail due to the order in which our blocks were completed (81%; *N* = 48/59) and sufficient dedicated time to study for Step 1 (85%; *N* = 50/59) (Table 2). A majority of students (59%; *N* = 35/59) agreed or strongly agreed that they were prepared for Step 1 being pass/fail due to the delivery of basic science content in class (i.e., lecturing style). In open responses, three students liked endocrinology as their last block, and two students commented that they had enough time to study during the dedicated Step 1 studying period.

Students were divided on whether they felt the aspect of in-house test questions similar to NBME-style prepared them for Step 1 being pass/fail, with 44% agreeing (*N* = 26/59) and 41% (*N* = 24/59) disagreeing (Table 2). However, of the thirty-seven open responses collected in the curriculum section of the survey, they do not reflect this divide; nineteen of the open responses expressed wanting more in-house NBME-style questions on weekly quizzes and for content in the curriculum to follow Step 1 tested concepts more closely. A divide was present when asked whether they agree or strongly agree that content taught in class helped prepare them for Step 1 being pass/fail (39%; *N* = 23/59) (Table 2). Sixteen students commented that the content taught in lectures could be more aligned with what was tested on Step 1. Lastly, there was a divide on whether there was enough time to study for Step 1 during Pillar 1, with 42% (*N* = 25/59) disagreeing and 49% (*N* = 29/59) agreeing (Table 2). Interestingly, in the mental health open-response section, 7% (*N* = 2/28) of students felt that they did not have sufficient time to study for Step 1 during Pillar 1.

### Academic Advising

The majority of students (67%; *N* = 44/66) agreed or strongly agreed that the school’s academic advising overall prepared them well for Step 1 being pass/fail. A substantial majority of students (80%; *N* = 47/59) agreed or strongly agreed that peer guidance/advice and “unofficial advice” from peers helped prepare them for Step 1 being pass/fail (Table 3). A majority of students (61%; *N* = 36/59) agreed or strongly agreed that structured student panels with Q&As helped prepare them for Step 1 being pass/fail (Table 3). A total of twenty-five open responses were collected from the academic advising section. Six students commented that they desired that upperclassmen who sat on the student panels represent the perspective of those who were challenged and those who performed well. Two students said that the upperclassmen advice was the best Step 1 guidance they received.

**Table 3:** Percentage of respondents who felt the following aspects of the school’s academic advising adequately prepared them for Step 1 being pass/fail out of fifty-nine responses

Academic advising categories	Strongly disagree	Disagree	Neutral	Agree	Strongly agree
Off the record peer guidance and “unofficial advice”	3.4	8.5	8.5	42.4	37.3
Structured student panels + Q&A with class of 2023 or 2024	8.5	17.0	13.6	45.8	15.3
Advising for exam readiness	0.0	6.8	27.1	59.3	6.8
1:1 meetings with the Medical Education Learning Specialist (MS2025) or Assistant Dean Medical Student Affairs (MS2024)	5.1	1.7	37.3	42.4	13.6
Tools to determine specialty fit	8.5	23.7	39.0	25.4	3.4
School-offered career advising sessions	6.8	10.2	39.0	35.6	8.5
Promoting resume competitiveness (research, extracurricular, and other opportunities)	5.1	13.6	30.5	37.3	13.6

A majority of students agreed that advising for exam readiness (66%; *N* = 39/59) and one-on-one meetings with a Learning Specialist (class of 2025) or Medical Student Affairs faculty (class of 2024) (56%; *N* = 33/59) prepared them for Step 1 being pass/fail (Table 3). The school had these predetermined faculty reach out to students before the Step 1 dedicated study period began and at least two times during dedicated study. The goal of the meetings was primarily to discuss Step 1 readiness based on previous practice exam scores or, in some cases, to develop a test preparation schedule and see how the student was doing overall. Student comments were overwhelmingly positive, with eighteen students mentioning how helpful faculty one-on-one meetings were, where they discussed concerns and exam preparation. Two students desired to have more regular faculty meetings than were originally scheduled. These open responses were gathered from both the mental health and academic advising sections.

## Discussion

Previous studies have individually evaluated mental health and curriculum perspectives for medical students since Step 1 has become pass/fail. Our study assessed student perspectives broadly in mental health, academic advising, and curriculum since Step 1 has moved to pass/fail within a 1.5-year pre-clerkship curriculum. As 62% of medical schools anticipate decreasing their pre-clerkship curriculum from the traditional 2 years to 1.5 years, with 21% of those schools feeling that Step 1 pass/fail influenced this decision, our results become relevant for those preparing for this shift in pre-clerkship curriculum [14]. Results of our study suggest students may benefit from increased and earlier introduction and integration of NBME-style test preparation within the official curriculum. Results suggest that incorporating a learning specialist to help students with individualized learning needs and increasing the meeting frequency beyond three required meetings with this faculty member may benefit student Step 1 outcomes in both mental health and exam readiness. Our results also suggest that one mental health counselor does not meet the demand for a student body of approximately 270 students. Schools with a similar student body size may consider hiring two counselors dedicated to their student body to better support the mental health of their students.

Only 48% of medical schools report integrating existing video lectures or question bank resources into the formal pre-clinical curriculum, making our results very applicable to over half of medical schools in the USA [14]. In our study, increased exposure to both NBME practice questions throughout Pillar 1 and NBME-style in-house exams was identified to enhance medical student preparation for Step 1 being pass/fail. Students’ comments were strongly in favor of re-examining in-house examination material, such as weekly quizzes, to align with NBME format and challenge level more closely.

Students at our school find peer advising valuable for Step 1 pass/fail preparation. Our results demonstrated that most of our surveyed medical students found Step 1 student panels, “unofficial advice,” and peer advising integral to their exam preparation. In the open responses, students desired a more diverse Step 1 student panel with individuals who would be able to represent both successes and challenges of the Step 1 pass/fail exam. Peer mentoring is an effective way to prepare medical students for the Step 1 exam [15, 16]. Upperclassmen support groups for second-year medical students were found to significantly increase Step 1 scores for students through both habit-building and resource advising [15]. Attendance at weekly near-peer tutoring sessions was also significantly correlated with students’ performance in overall basic sciences, CBSE mid-year, CBSE final, and USMLE Step 1 examinations [16]. Incorporating structured peer mentoring specifically for Step 1 may be an easy way to help students improve exam outcomes.

Step 1 advising meetings between medical students and school faculty and direct access to a mental health counselor were helpful for student preparedness and mental health since the exam became pass/fail. Our institution appointed one faculty member to the class of 2024 and 2025 to see how students are doing overall and advise with goal-directed exam preparation and readiness checks for students before and during their dedicated period. Faculty-led advisor meetings may help students identify individualized coping strategies to mitigate stress and anxiety during Step 1 preparation [17]. In our study, students from both classes emphasized in both the data and open responses the helpfulness of faculty-led advisor meetings and Learning Specialist meetings for predicting exam readiness and overall support. In the open responses section of advising, 72% (*N* = 18/25) of students mentioned the helpfulness of the meetings with the Learning Specialist or the faculty-led advisor meetings. Other schools may consider hiring an individualized Learning Specialist for Step 1 readiness. If a Learning Specialist is already hired, they may consider increasing the frequency of meetings and beginning these meetings earlier in the pre-clerkship curriculum.

Students who met with a medical student counselor found it to be supportive of their mental health, with one student noting a tough time scheduling an appointment due to the high demand of the counselor’s time. These results suggest our medical school would benefit from hiring an additional mental health counselor for a total of two for our student body of approximately 270 students. Fewer than 14% of schools fully adhere to all established AAMC guidelines for mental health [18, 19]. Medical school adherence to these guidelines is expected to be reported in the school-specific policies and procedures manuals or handbooks. Further, 25% of schools do not have published documentation for the availability of confidential counseling services for medical students [19]. These findings demonstrate that medical schools at large may need to enhance their policies and procedures handbooks for medical students and ensure they are at a minimum adhering to the AAMC guidelines to provide infrastructure for students in need of mental health resources.

In addition to the unmet student needs of increasing access to mental health counselors and improving student handbooks, both structured and unstructured socialization have been shown to improve mental health for medical students preparing for Step 1 [16, 17]. Results from our study strongly suggest the benefit of unofficial or unstructured advice from peers and structured student panels to help students prepare for Step 1 being pass/fail. Student-led, structured exam preparation sessions for medical students reduce Step 1 stress and anxiety [20]. Effective student-led topics of discussion after entering the second year of medical school include Step 1 test-taking strategies, planning and preparation for protected Step 1 study time, and post-test anxiety [20]. Unstructured socialization, such as seeing friends and family, is one of the common coping strategies of medical students preparing for Step 1 exams [17] and may help improve mental health during the Step 1 study period. Increasing student-led preparation and encouraging time with a student’s social circle are ways to increase the mental health of students preparing for Step 1.

Results from our study may not provide the full perspectives needed for schools looking to implement change. The responses from the survey suggest a wide range of student perspectives, but we are unsure how students performed on their exam or if our results represent those who did not pass Step 1 on their first attempt, which may make the results of this study difficult to generalize. Our study cannot control for response or recall bias, as the survey was not collected immediately following the students’ Step 1 examination dates. We did not have a cohort that took Step 1 for a numerical score and therefore do not have a direct comparison for those who took the exam pass/fail. Finally, the survey was specific to our school’s medical education and may lack generalizability to other medical schools. For example, our school completes pre-clerkship curriculum in 1.5 years, whereas 6% of medical schools end the pre-clerkship phase after 1 year, 29% end it after 1.5 years, and 56% end it after 2 years [21].

## Conclusion

Although medical school leadership may feel they have a grasp on student needs, qualitative and quantitative data after Step 1 transitioned to new Step 1 pass/fail scoring is worth collecting to evolve medical education. Our results may be most relevant to the numerous medical schools anticipating decreasing their preclinical curriculum from 2 years to 1.5, helping them shape their 1.5-year pre-clerkship medical education to the unique demands of Step 1 pass/fail. Our study demonstrates areas for improvement at our medical school and highlights unmet mental health and curriculum student needs at other schools, such as incorporating outsourced resources into the official curriculum and assessing if the student handbook for policies and procedures aligns with AAMC guidelines for mental health. Future directions for research include medical schools with a 1.5-year pre-clerkship curriculum soliciting a similar survey to assess their own medical education’s impact on students with Step 1 now being pass/fail.

## Supplementary Information

Below is the link to the electronic supplementary material.

Supplementary file1 (DOCX 35 KB)

## Data Availability

Data is available upon request.
