# Supplementary material for: Strategies for Success: Assessing Student Perspectives on the Impact of Step 1 Pass/Fail
Source: Med Sci Educ. 2025 Jun 18;35(5):2343–50. doi: 10.1007/s40670-025-02434-4 (PMC12812120; doi:10.1007/s40670-025-02434-4)
Supplement: Supplementary file 1 — Supplementary file1 (DOCX 35 KB) [file 40670_2025_2434_MOESM1_ESM.docx]

Title: Strategies for Success: Assessing Student Perspectives on the Impact of Step 1 Pass/Fail

Medical Science Educator

Michaela Derby, Callie Olson, Pasquale Manzerra, Lori Hansen

University of South Dakota Sanford School of Medicine

Corresponding author email: Michaela.j.derby@gmail.com

**Supplementary Information 1:**

These are the survey questions emailed to students in the class of 2024 and 2025. The survey was accessed through an embedded hyperlink in an email sent to students explaining the purpose, time to complete, anonymity, and that informed consent was implied, if completed. We surveyed students who completed Step-1 primarily in January 2022 and 2023. The first 1.5 years of curriculum is known as Pillar 1 at our school. The survey was sent to every medical student in the class of 2024 (n=62) and 2025 (n=63), and all were eligible to participate. The survey was initially sent September 11, 2023 and we accepted responses until September 29, 2023. Emails containing the survey hyperlink were sent a total of four times.

Step 1 Pass/Fail Survey

Start of Block: Demographics

Q1 What year will you graduate?

Graduation Year

- 2024
- 2025

Q5 When did you take Step 1?

________________________________________________________________

Q7 What gender do you identify with?

- Male
- Female
- Non-binary / gender fluid
- Other
- Prefer not to say

End of Block: Demographics

Start of Block: Question 1: Overview

Q8 The school prepared you well for Step 1 being pass/fail in the following areas:

|  | 1 - strongly disagree | 2 - disagree | 3 - neutral | 4 - agree | 5 - strongly agree |
| --- | --- | --- | --- | --- | --- |
| Mental Health |  |  |  |  |  |
| Curriculum |  |  |  |  |  |
| Academic Advising |  |  |  |  |  |

End of Block: Question 1: Overview

Start of Block: Question 2: Mental Health

Q9 The school adequately supported your mental health with Step 1 being pass/fail in the following areas:

|  | 1 - strongly disagree | 2 - disagree | 3 - neutral | 4 - agree | 5 - strongly agree |
| --- | --- | --- | --- | --- | --- |
| Counseling services |  |  |  |  |  |
| Advising about wellness |  |  |  |  |  |
| Wellness Committee events |  |  |  |  |  |
| Other: |  |  |  |  |  |

Q10 What else would you like to share about mental health regarding Step 1 being pass/fail?

________________________________________________________________

End of Block: Question 2: Mental Health

Start of Block: Curriculum

Q11 The following aspect of the school’s curriculum adequately prepared you for Step 1 being pass/fail:

|  | 1- strongly disagree | 2 - disagree | 3 - neutral | 4 - agree | 5 - strongly agree |
| --- | --- | --- | --- | --- | --- |
| Weekly quizzes |  |  |  |  |  |
| Completing pillar 1 in 1.5 years |  |  |  |  |  |
| Test question similarity to NBME style questions |  |  |  |  |  |
| Completing CBSEs after Medical Foundations 2, Blood block, and Endocrine block |  |  |  |  |  |
| Completing a NBME after each block |  |  |  |  |  |
| Integrated clinical skills into pre-clerkship curriculum |  |  |  |  |  |
| Order in which the blocks were completed (i.e., MF1 > MF2 > Skin/Msk > neuro) |  |  |  |  |  |
| Content taught in class versus content tested on Step 1 |  |  |  |  |  |
| Delivery of Basic Science content in class (i.e. lecturing styles) |  |  |  |  |  |
| Sufficient time during Pillar 1 to study for Step 1 |  |  |  |  |  |
| Sufficient dedicated time to study for Step 1 |  |  |  |  |  |
| Supplemental Instruction Review Sessions |  |  |  |  |  |
| Other: |  |  |  |  |  |

Q12 What else would you like to share about the curriculum regarding Step 1 being pass/fail?

________________________________________________________________

End of Block: Curriculum

Start of Block: Academic Advising

Q13 The following aspect of the school’s academic advising adequately prepared you for Step 1 being pass/fail:

|  | 1 - strongly disagree | 2 - disagree | 3 - neutral | 4 - agree | 5 - strongly agree |
| --- | --- | --- | --- | --- | --- |
| Off the record peer guidance and “unofficial advice” |  |  |  |  |  |
| Structured student panels + Q&A with class of 2023 or 2024 |  |  |  |  |  |
| Advising for exam readiness |  |  |  |  |  |
| 1:1 meetings with the Medical Education Learning Specialist (MS2025) or Assistant Dean Medical Student Affairs (MS2024) |  |  |  |  |  |
| Tools to determine specialty fit |  |  |  |  |  |
| School-offered career advising sessions |  |  |  |  |  |
| Promoting resume competitiveness (research, extracurricular, and other opportunities) |  |  |  |  |  |
| Other: |  |  |  |  |  |

Q14 What else would you like to share about advising in regard to Step 1 being pass/fail?

________________________________________________________________

End of Block: Academic Advising

Start of Block: Preparation

Q15 How much time did you spend preparing for Step 1 during the Pillar 1 summer? (hours per week)

|  | 0 | 5 | 10 | 15 | 20 | 25 | 30 | 35 | 40 |
| --- | --- | --- | --- | --- | --- | --- | --- | --- | --- |

| Hours | 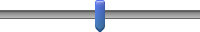 |
| --- | --- |

Q16 If you were to redo your Step 1 preparation, how much would you have studied in the summer per week? (hours)

|  | 0 | 4 | 8 | 12 | 16 | 20 | 24 | 28 | 32 | 36 | 40 |
| --- | --- | --- | --- | --- | --- | --- | --- | --- | --- | --- | --- |

| Hours | 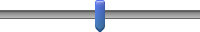 |
| --- | --- |

Q17 How would you describe your study tactics for Step 1 during the Fall semester prior to taking Step 1?

- A consistent number of hours each week
- Ramped up in hours or questions each week
- Inconsistent
- None

Q18 How much did the following priorities negatively impact your motivation to study for Step 1 during the Fall semester prior to your exam date? (Not at all, slightly, moderately, very much, extremely so)

|  | Not at all | slightly | moderately | very much | extremely so |
| --- | --- | --- | --- | --- | --- |
| Step 1 being pass/fail rather than scored |  |  |  |  |  |
| Class rank |  |  |  |  |  |
| Focusing on achieving good grades |  |  |  |  |  |
| Focusing on understanding content for each block |  |  |  |  |  |
| Focusing on receiving a passing grade |  |  |  |  |  |
| Increasing extracurriculars |  |  |  |  |  |
| Spending time with family |  |  |  |  |  |
| Maintaining good mental health |  |  |  |  |  |

Q19 How many hours did you spend each week studying for Step 1 during each block period? (1-10+)

|  | 0 | 1 | 2 | 3 | 4 | 5 | 6 | 7 | 8 | 9 | 10 |
| --- | --- | --- | --- | --- | --- | --- | --- | --- | --- | --- | --- |

| Cardiovascular | 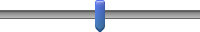 |
| --- | --- |
| Pulmonology | 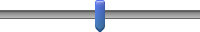 |
| Renal | 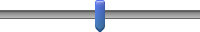 |
| Endocrinology | 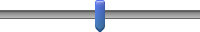 |

Q20 In retrospect, how many hours per week during the blocks do you wish you had completed each week to adequately prepare for Step 1? (1-10+)

|  | 0 | 1 | 2 | 3 | 4 | 5 | 6 | 7 | 8 | 9 | 10 |
| --- | --- | --- | --- | --- | --- | --- | --- | --- | --- | --- | --- |

| Cardiovascular | 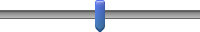 |
| --- | --- |
| Pulmonology | 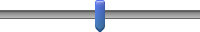 |
| Renal | 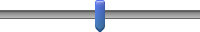 |
| Endocrinology | 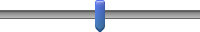 |

Q21 Did you find certain blocks easier, or harder to study for Step 1 during? If so, please explain.

________________________________________________________________

Q22 In your own words, what do you think was the biggest factor impacting how much you studied for Step 1 during the Fall 2022 Semester?

________________________________________________________________

Q23 Would you be willing to participate in a low time commitment working group to discuss your thoughts on your Step 1 pass/fail study experience?

- Yes
- No

End of Block: Preparation
